# Automatic biomarker discovery and enrichment with BRAD

**DOI:** 10.1093/bioinformatics/btaf159

**Published:** 2025-05-05

**Authors:** Joshua Pickard, Ram Prakash, Marc Andrew Choi, Natalie Oliven, Cooper Stansbury, Jillian Cwycyshyn, Nicholas Galioto, Alex Gorodetsky, Alvaro Velasquez, Indika Rajapakse

**Affiliations:** Gilbert S. Omenn Department of Computational Medicine and Bioinformatics, University of Michigan Medical School, Ann Arbor, MI 48109, United States; Gilbert S. Omenn Department of Computational Medicine and Bioinformatics, University of Michigan Medical School, Ann Arbor, MI 48109, United States; Department of Mathematics, University of Michigan, Ann Arbor, MI 48109, United States; Gilbert S. Omenn Department of Computational Medicine and Bioinformatics, University of Michigan Medical School, Ann Arbor, MI 48109, United States; Gilbert S. Omenn Department of Computational Medicine and Bioinformatics, University of Michigan Medical School, Ann Arbor, MI 48109, United States; Gilbert S. Omenn Department of Computational Medicine and Bioinformatics, University of Michigan Medical School, Ann Arbor, MI 48109, United States; Department of Biomedical Engineering, University of Michigan, Ann Arbor, MI 48109, United States; Gilbert S. Omenn Department of Computational Medicine and Bioinformatics, University of Michigan Medical School, Ann Arbor, MI 48109, United States; Department of Aerospace Engineering, University of Michigan, Ann Arbor, MI 48109, United States; Department of Computer Science, University of Colorado Boulder, Boulder, CO 80309, United States; Gilbert S. Omenn Department of Computational Medicine and Bioinformatics, University of Michigan Medical School, Ann Arbor, MI 48109, United States; Department of Mathematics, University of Michigan, Ann Arbor, MI 48109, United States

## Abstract

**Motivation:**

Integrating Large Language Models (LLMs) with research tools presents technical and reproducibility challenges for biomedical research. While commercial artificial intelligence (AI) systems are easy to adopt, they obscure data provenance, lack transparency, and can generates false information, making them unfit for many research problems. To address these challenges, we developed the Bioinformatics Retrieval Augmented Digital (BRAD) agent software system.

**Results:**

Here, we introduce BRAD, an agentic system that integrates LLMs with external tools and data to streamline research workflows. BRAD’s modular agents retrieve information from literature, custom software, and online databases while maintaining transparent protocols to increase the reliability of AI generated results. We apply BRAD to a biomarker discovery pipeline, automating both execution and the generation of enrichment reports. This workflow contextualizes user data within the literature, enabling a level of interpretation and automation that surpasses conventional research tools. Beyond the workflow we highlight here, BRAD is a flexible system that has been deployed in other applications including a chatbot, video RAG, and analysis of single cell data.

**Availability and implementation:**

The source code for BRAD is available at https://github.com/Jpickard1/BRAD; Information for pip installation, tutorials, documentation, and further information can be found at: ReadTheDocs.

## 1 Introduction

From automating experiments (Boiko *et al.* 2023) to enabling virtual laboratories (Swanson *et al.* 2024), artificial intelligence (AI) and large language models (LLMs) are transforming biomedical research workflows. LLMs’ ability to integrate unstructured information makes them a promising tool for interpreting experimental data based upon existing scientific knowledge (Schaefer *et al.* 2024). For instance, discovering new marker genes and understanding their biological significance often requires manually contextualizing findings within published knowledge—a process that is both time-intensive and prone to human inconsistencies. The repetitive nature of this task and the extensive tools already developed for this purpose make it an ideal candidate for agentic automation (Kuleshov *et al.* 2016, Luebbert and Pachter 2023). However, successfully integrating such systems into scientific workflows requires more than mere tool connectivity—it demands traceability, reproducibility, and verifiability of AI-generated results (Shavit *et al.* 2023).

Systems like ChatGPT and Perplexity AI combine user queries with tool readouts (e.g. web searches) before generating responses. Yet, users lack awareness into the origin, content, and reliability of retrieved information. These chatbots use Retrieval Augmented Generation (RAG) and agentic architectures, enabling AI models to interact with tools and access external information (Lewis *et al.* 2020). Open-source implementations improve data provenance and reproducibility over commercial tools, and agentic and RAG systems reduce hallucinations (Craig and Drăghici 2024, Matsumoto *et al.* 2024, 2025).

The Bioinformatics Retrieval Augmented Digital (BRAD) agent was developed to address challenges of reproducibility while maintaining flexibility to integrate into a wide array of scientific workflows. This system connects LLMs to bioinformatics tools including online databases, research literature, and custom software. Designed for flexibility and ease of use, BRAD requires no advanced setup and allows integration of new tools and databases. BRAD’s transparent use of LLMs and research tools enhance the system’s verifiability, making it suitable for real-world research tasks. The remainder of this article details BRAD’s implementation and its application to automating a biomarker pipeline and generating enrichment analysis report based on data and research literature.

## 2 Software architecture

A BRAD Agent—a virtual AI system with processing, memory, and interaction capabilities—orchestrates interactions between the user, a LLM, and several tools. Connecting Agents to research tools improves the reliability of AI generated outputs. We outline the core structure and several research tools the Agents have below.

### 2.1 BRAD Agent class

**Figure 1.**
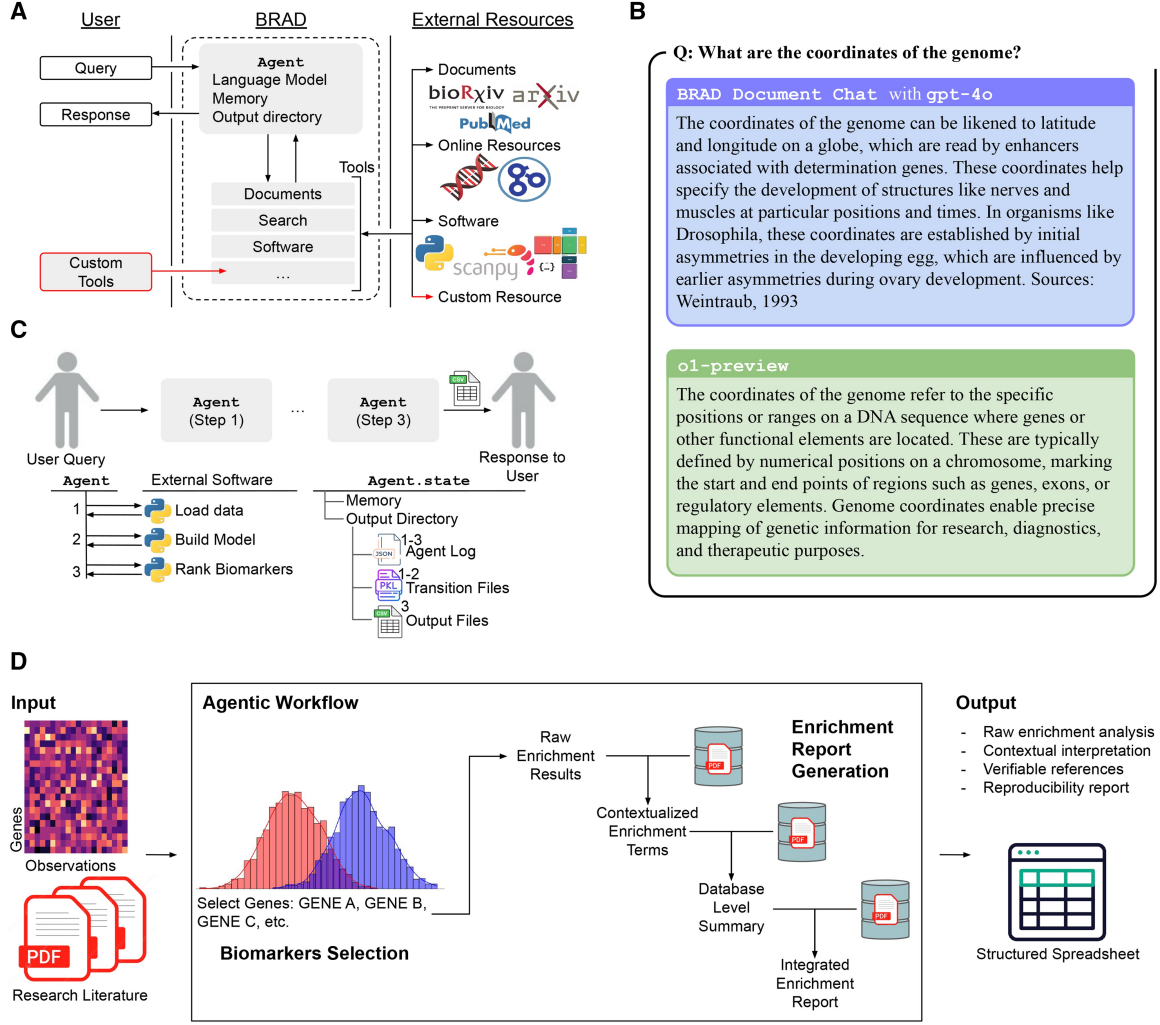
Overview of software and example workflow. (A) The software uses the Agent as an interface between the user, the LLM, and tools that access external resources. (B) Example outputs from a LLM versus BRAD’s Document Chat system. (C) Agentic workflows coordinate the use of multiple tools and save intermediate steps and reproducibility information to the Agent’s output directory. (D) BRAD’s workflow automates gene enrichment analysis of RNA sequencing data and contextualizes enrichment results through the generation of a report via chain of thought and verifiable references to the user’s documents.

The Agent class serves as BRAD’s user interface and is responsible for managing memory, LLM utilization, planning, and data organization within a dedicated output directory (Fig. 1A). This class is built upon LangChain, and it maintains detailed logs of all interactions and decisions to ensure transparency and reproducibility (Affarth 2023) (Supplementary Table S3). Each interaction with the Agent generates an entry in a JSON log file containing:

‘PROMPT’ : {input from the user}

‘OUTPUT’ : {output to the user}

‘LLM’ : {API connection, model, and settings}

‘PROCESS’: {chain of thought for how the tool was used}

‘SOURCES’: {list of referenced information used to

generate LLM response}

These and other data are recorded to ensure transparency in the Agent’s decision-making. Each Agent can be customized with different settings from configurations file and can be ran locally or use any LLM hosted by NVIDIA, OpenAI. Further configurations, such as search and database options, can be customized for each tool module.

### 2.2 Document chat tool

The Document Chat module retrieves information from documents, including research articles or any PDF, using a RAG workflow. The retrieved information is incorporated into a prompt template, such as the following, which is then passed to the LLM (Sahoo *et al.* 2024):

System: Respond to the human based on the

provided sources.

Sources: {Retrieved and augmented text is placed here.}

Human: {User input is placed here.}

Providing explicit, well-sourced information to the LLM prompt improves the generated response by anchoring replies in reliable information and allowing users to verify the response against cited, scientific literature (Lewis *et al.* 2020).

To illustrate use of this tool, consider the question: What are the coordinates of the genome? Answered in Fig. 1B, this open ended question is subject to several interpretations. While several answers are scientifically valid, the BRAD’s answer has the advantage of providing a verifiable reference for its source (Weintraub 1993).

BRAD’s Document Chat tool enables the Agent to search thousands of papers that are selected by a user. When constructing an Agent, users choose configurations for the number of documents, retrieval mechanisms, and information processing of the RAG pipeline. This tool is benchmarked in Supplementary Information Section S1.3. Here we focuses on implementation rather than methodological advancements, so we refer readers to Gao *et al.* (2023) for a survey on RAG systems.

### 2.3 Database search tool

The Database Search tool retrieves information from online, external databases. BRAD can search for literature on arXiv, bioRxiv, and PubMed, and pull information and data from Enrichr and Gene Ontology. Custom prompt templates facilitate literature and database searches by leveraging the LLM to select the appropriate database and search terms. The system can take user queries and identify search terms, or it can select data from files. An example of the prompt template is as follows:

System: Select an appropriate database for the user’s query.

Database: {ARXIV, ENRICHR, …}

Human: {User input is placed here.}

**Output:**

1. < Enter a database name >

2. < Enter search terms >

After searching information from the online resources, an Agent uses the LLM to respond to the user query.

### 2.4 Graphical user interface (GUI)

The BRAD GUI offers a chatbot interface for interacting with an Agent. The underlying architecture includes a Node.js frontend that communicates with a Flask API server built atop of the BRAD Python package. The Flask API, contains endpoints to/invoke the Agent class and query the BRAD chatbot; create, delete, and manage chatbot/sessions and RAG/databases; select the usage of different/llms; and manipulate different settings or/configurations.

The system can also be integrated by installing BRAD via pip, enabling an Agent to interact directly with a research software pipeline. The Agent and its tools and interface improve the reliability of AI generated outputs, making the BRAD system deployable in real workflows.

## 3 Automatic biomarker discovery and report generation

We deployed BRAD to generate a report that analyzes experimental data in the context of research literature. This workflow takes from a user gene expression (RNA-seq) data and research literature as input, performs both biomarker selection and enrichment analysis, and produces a report interpreting the data (Fig. 1D).

### 3.1 Software tool performs biomarker discovery

BRAD’s Software Tool module allows the Agent to execute and retrieve information from software pipelines, external to the BRAD system. The Agent selects and executes the appropriate software based on the user’s request and documentation retrieved from external software. The output of the external software along with the user instructions are passed through the LLM to respond to the user. The Software Tool can execute any Python codes that can be found from the Agent configuration file.

The multistage biomarker identification workflow of Pickard *et al.* (2024) requires the Agent to run several software scripts for structuring, modeling, and performing observability analysis. To initiate this, the user prompts an Agent:

User: Perform biomarker selection on:

< path/to/data > /genes.h5ad

In this pipeline, the dataset genes.h5ad is stored according to a widely used standard required by the external software pipeline. To carry out this workflow from a single user query, the Agent manages a queue of actions, using the output directory and internal memory to transfer information between stages (Fig. 1C). The output of this process is a spreadsheet generated by the software, structured as follows:

| Biomarker | λ | Rank |

------------------------

| CDT1 | 10 | 1 |

| PCNA | 7 | 2 |

Because the Agent records the interactions of all LLM and external tool, the result is entirely reproducible. These biomarkers serve as input to the next stage of this workflow.

### 3.2 Chain of thought gene enrichment

The Agent utilizes its other tool modules to generate a report that interprets the biological roles of the biomarkers within the context of the user’s literature. First, biomarkers are enriched to identify biological pathways, cell types, and ontology terms. Then, multiple layers of Chain of Thought (CoT) reasoning, combined with the Document Chat, refine these results by providing verifiable references for the Agent’s interpretation of the data (Wei *et al.* 2022) (Fig. 1D). The BRAD Agent cross-references enrichment results with the user’s literature knowledge base to explain identified terms and interpret findings across different enrichment databases. These insights are synthesized into a high-level interpretation, highlighting the biological significance of the biomarker gene set and providing an explanation of the user’s data in context of literature tailored for the study.

The Agent automates this process while maintaining a high degree of transparency and verifiability. Each section of the report includes a plain-text CoT interpretation alongside relevant references and quotations from the literature. This allows the user to validate each of the Agent’s interpretations against the user’s custom knowledge base. This transparency improves the automation without sacrificing the rigor of the report.

### 3.3 Results

This workflow produces an Excel workbook containing both analysis and documentation to ensure transparency and reproducibility. The report includes a high-level results summary, enrichment analysis results with Agent interpretations for each database, and a reproducibility report. We applied this workflow to a dataset investigating cellular proliferation, along with literature from our digital library. Biomarker genes were identified from bulk RNA-seq data collected from eight populations of synchronized human fibroblasts (Chen *et al.* 2015). The rankings from the first stage were reviewed and refined before being analyzed using the CoT enrichment report workflow. The high-level CoT interpretation in the report captured key aspects of the data, including gene ontology terms such as RNA processing (GO:0006397), splicing (GO:0000398, GO:0000377), and gene expression (GO:0010467). It also identified pathways relevant to cell cycle regulation (Supplementary Information Section S1.1.1, Supplementary File S1).

To assess the accuracy of literature retrieval in the CoT enrichment workflow, we constructed a test database comprising 30 papers equally pulled from biology, mathematical biology, and computer science literature. We then generated ten reports analyzing using genes involved in the cell cycle. Of all references (*n*=1069) in the reports produced by the Agent, 92.61% of citations were to biological literature, and 82.20% of all quotations passed through the LLM were unique. This suggests that the Agent effectively filtered out unrelated sources while maintaining a diverse selection.

To evaluate how well the Agent identifies relevant biological terms, we deployed three Agents: one using only a LLM (equivalent to a commercial chatbot without any tools), one with the Document Chat tool, and one utilizing the CoT enrichment workflow. Each Agent was given gene sets (n=886) and tasked with identifying relevant biological terms. The responses were then compared against the ground truth from which the gene sets were originally derived, and performance was assessed based on how well the generated terms matched the ground truth enrichment terms (Table 1, Supplementary Information Section S1.1.2). The Agent using only the LLM always generated a response, while the Agent with the Document Chat tool often indicated insufficient information based on available literature references. In contrast, the Agent utilizing the CoT enrichment workflow consistently performed best at identifying cell types, pathways, and ontology terms. This aligns with the expectation that the Agent equipped with enrichment database access outperforms LLM systems that lack integration with research tools. While the improved enrichment results from the fully equipped BRAD Agent are promising, the true value of this workflow lies in its ability to automate report generation based on the accuracy of the Agent’s output—a task that is otherwise performed manually.

**Table 1.** Agentic enrichment.^a^

Dataset	Agent	Agent with document chat	Agent with CoT workflow
Ontology terms	37.39±23.47	37.51±19.89	**80.24 ± 15.29**
Pathways	61.68±39.48	49.50±36.71	**99.08 ± 07.17**
Cell types	28.89±19.44	28.42±17.18	**90.59 ± 18.46**

a The table presents the Normalized Token Indel Similarity (0–100) between the enrichment term and the Agent’s response. Bold values indicate the highest similarity.

This end-to-end workflow streamlines biomarker analysis while ensuring transparency in data and AI utilization. The Agent automates biomarker discovery by integrating external software and logging each action. During report generation, it synthesizes LLM and database readouts, enabling automated interpretation of data in context of a custom knowledge base. This provides an automated AI solution that supports human interpretation of the data while maintaining reproducibility and verifiability.

## 4 Summary

This article introduces BRAD, a prototype AI Agent that integrates LLMs with research workflows, while addressing challenges of reproducibility and transparency required to deploy a tool in a scientific environments. BRAD is designed to be both extensible and easy to deploy, making it an adaptable research tool capable of automating and integrating across a broad range of workflows. The Software Tool, for instance, has been deployed to a wide array of tasks, ranging from data visualization to generating embeddings from foundation models (examples found on Github). The utilization of agentic AI systems like BRAD is well suited for many repetitive research workflows beyond biomarker discovery and enrichment analysis. For example, we leveraged a BRAD Agent and the GUI API to develop an application for interacting with and interpreting decades of research seminar recordings, and we integrated an Agent to help visualize and exploring network data. These and other applications of BRAD require minimal coding to deploy an Agent and maintain complete transparency, making it a valuable tool for researchers.

## Supplementary Material

btaf159_Supplementary_Data
