# Supplementary material for: Automatic biomarker discovery and enrichment with BRAD
Source: Bioinformatics. 2025 May 5;41(5):btaf159. doi: 10.1093/bioinformatics/btaf159 (PMC12064167; doi:10.1093/bioinformatics/btaf159)
Supplement: btaf159_Supplementary_Data [file btaf159_supplementary_data.zip › BRAD_Bioinformatics_Final_SI.pdf]

## 1. Supplementary information

This section presents extended results and details related to the use cases of BRAD discussed in the paper.

### 1.1. Extended Results: Automatic Biomarker Discovery and Report Generation

#### 1.1.1. Supplementary File S1.

The report discussed in section §3.3 is provided as Supplementary File 1. This file may be accessed here: https://docs.google.com/spreadsheets/d/197jwa5flekKGdCt_Js7DHHKzuL_etG_L/edit?usp=sharing&ouid=105092191618690961896&rtpof=true&sd=true. This report is generated by passing selected biomarker genes through the CoT workflow described in §3.2. Prior to analysis, the biomarkers selected in §3.1 can be manually reviewed. This human checkpoint improves transparency, and the option to further tailor analysis, for example, by removing mitochondrial genes or selecting a particular number of biomarker genes. The report also includes a reproducibility page, which details the exact biomarkers used, the LLM, human input, and other relevant information to facilitate reproducing the AI-generated document. Further details regarding how enrichment is performed, Document Chat tool configurations, and utilization of the data can be found on the associated Github page.

#### 1.1.2. Evaluation of Identified Biological Terms

To evaluate the effectiveness of the CoT workflow in generating gene enrichment reports, we constructed three distinct `Agent` configurations:

- `Agent0`: This agent operated solely using the LLM (`gpt-3.5-turbo-0125`) without any additional tool modules.
- `Agent1`: This agent incorporated the document chat tool, granting it access to a curated database of 500 research papers available at https://rajapakse.lab.medicine.umich.edu/papers/digital-library.
- `Agent2`: This agent leveraged the full CoT workflow while accessing a database similar to that used by Agent1.

For Agent2, the final response was directly derived from the CoT-generated output. All agents utilized the same LLM (`gpt-3.5-turbo-0125`) to ensure consistency in model performance across conditions.

**Datasets.** To select genesets with ground truths, we used pedigreed datasets from Enrichr, including: KEGG Pathways (2021, human), Gene Ontology Terms (2021), and Tabula Sapiens. These data can be accessed here: https://maayanlab.cloud/Enrichr/#libraries

Each dataset had between 80 and 100 enrichment terms and their associated gene sets sampled. Each gene set was sampled to create 3 subsets, each with a maximum length of 80% or 20 genes. The subsets were created independently so that some genes are present in multiple subsets. To investigate enrichment for cell types, pathways, and ontology terms, we used the Tabula Sapiens, KEGG 2021 Human, and GO Biological Process 2023 datasets [Consortium* et al., 2022, Kanehisa and Goto, 2000, Ashburner et al., 2000].

**Experiment.** The gene sets and enrichment terms form pairs of input and target output from the three `Agent`s. The three agents were given the following prompts:

```
Agent0: "What enrichment_types are highly enriched based on the following gene list? Gene list=genes"

Agent1: """What enrichment_types are highly enriched based on the following gene list?
   **INSTRUCTIONS** You must provide an answer or guesses if you cannot find an answer in the referenced text. If you
guess, please state it is not indicated in the text and add several guesses to answer the question.
   Gene list=genes"""

Agent2: "What enrichment_types are highly enriched based on the following gene list?"
```

Initially, `Agent0` and `Agent1` were provided identical prompts. In many instanced, however, the Document Chat tool prevented `Agent1` from providing an answer, if the retrieved text was not sufficient to answer the question. While this guardrail is useful in many cases, for the purpose of our experiment, we chose to encourage `Agent1` to respond, even if no information is retrieved. The prompt for `Agent2` does not include the gene list because the genes list enters the workflow separately from the human query.

Each `Agent` generated a response to the above prompt for each of the gene sets ($n = 886$) using their respective tools and workflows. `Agent0` and `Agent1` provide a text response as a python string. To compare the output of `Agent2` with the other systems, we extract the highest level of the Chain of Thought text contained in the automatically generated Excel spreadsheet. We evaluate the performance of each `Agent` according to how each response identifies with the ground truth enrichment term for the gene sets that were entered into the prompt or workflow.

**Evaluation.** The responses produced by each `Agent` were compared with the target enrichment term according to the following metrics:

- **Normalized Token Indel Similarity** (NTI) is a similarity metric bounded between 0 and 100, and defined by:

$$NTI(\text{target}, \text{response}) = \sum_{x \in \text{target}} \min_{y \in \text{response}} \left(1 - \frac{\text{Levenshtein Distance}(x, y)}{\text{length}(x) + \text{length}(y)}\right).$$

| Dataset | Target Length | Agent0 | Agent1 | Agent2 |
|---|---|---|---|---|
| Ontology Terms | 51.5 | $25.10 \pm 8.34$ | $26.00 \pm 8.36$ | $13.03 \pm 4.61$ |
| Pathways | 25.4 | $07.26 \pm 8.19$ | $09.73 \pm 8.18$ | $01.26 \pm 1.54$ |
| Cell Types | 24.9 | $13.38 \pm 6.87$ | $14.17 \pm 7.08$ | $02.41 \pm 3.74$ |

**Table S1.** Token Levenshtein Distance between the `Agent` responses and target terms.

Here, $x$ and $y$ are individual words found within the target and response strings. Good responses have a high NTI similarity. Table 1 within the main text compared the performance of the `Agent`s according to NTI similarity.

- **Token Levenshtein Distance** this compares the two strings based upon the minimum number of single-character edits required to turn each word in the target enrichment type into a word in the `Agent`'s response [Levenshtein, 1966]

$$\text{Levenshtein Distance(target, response)} = \sum_{x \in \text{target}} \min_{y \in \text{response}} \big(\text{Levenshtein Distance}(x, y)\big)$$

Good responses have a small Levenshtein Distance. Table S1 compared the performance of the `Agent`s according to Token Levenshtein Distance.

Both metrics indicate a strong propensity for `Agent2` to deliver responses that are more likely to contain the ground truth enrichment term. This suggest a reasonably high degree of accuracy in the chain of thought enrichment workflow.

The utility of `Agent2`'s workflow extends beyond improved performance metrics to the automation it provides in generating reports. While there are no established metrics to compare the effectiveness of agentic automation against human performance, its value has become evident through real-world data analysis and its utilization as a tool. We have developed a command line tool, which can be accessed from the Github page, to improve access to utilize this particular workflow.

## 1.2. Benchmarking Tool Modules

This section profiles the use of the LLM in different modules of BRAD. The major cost to the user occurs in terms of (1) runtime and (2) API fees. There is no substantial memory requirements for this system. A user could also elect to run the LLM inference locally, in which case there would be no fees and the time requirements depend on the user's hardware. The following metrics are evaluated:

- Total Time: the time required to respond to the users query by a single module.
- LLM Time: the time required for the LLM to perform its task within the module.
- Total Cost: the total cost of LLM API fees within a single module. This can represent multiple LLM utilizations.
- LLM Cost: the cost of LLM API fees for the LLM to perform its task within a module.
- Input Tokens: the number of tokens input for a single LLM query.
- Output Tokens: the number of tokens output for a single LLM query.
- Total Tokens: the sum of input and output tokens for a single LLM query.

We measured these seven metrics for user queries requiring BRAD to perform different tasks: engaging in question-answering (i.e., functioning purely as a chatbot), searching online databases (which involves selecting a database and potentially loading data from a file), and interacting with software by reading and writing software code and documentation. See fig. S1 and table S2.

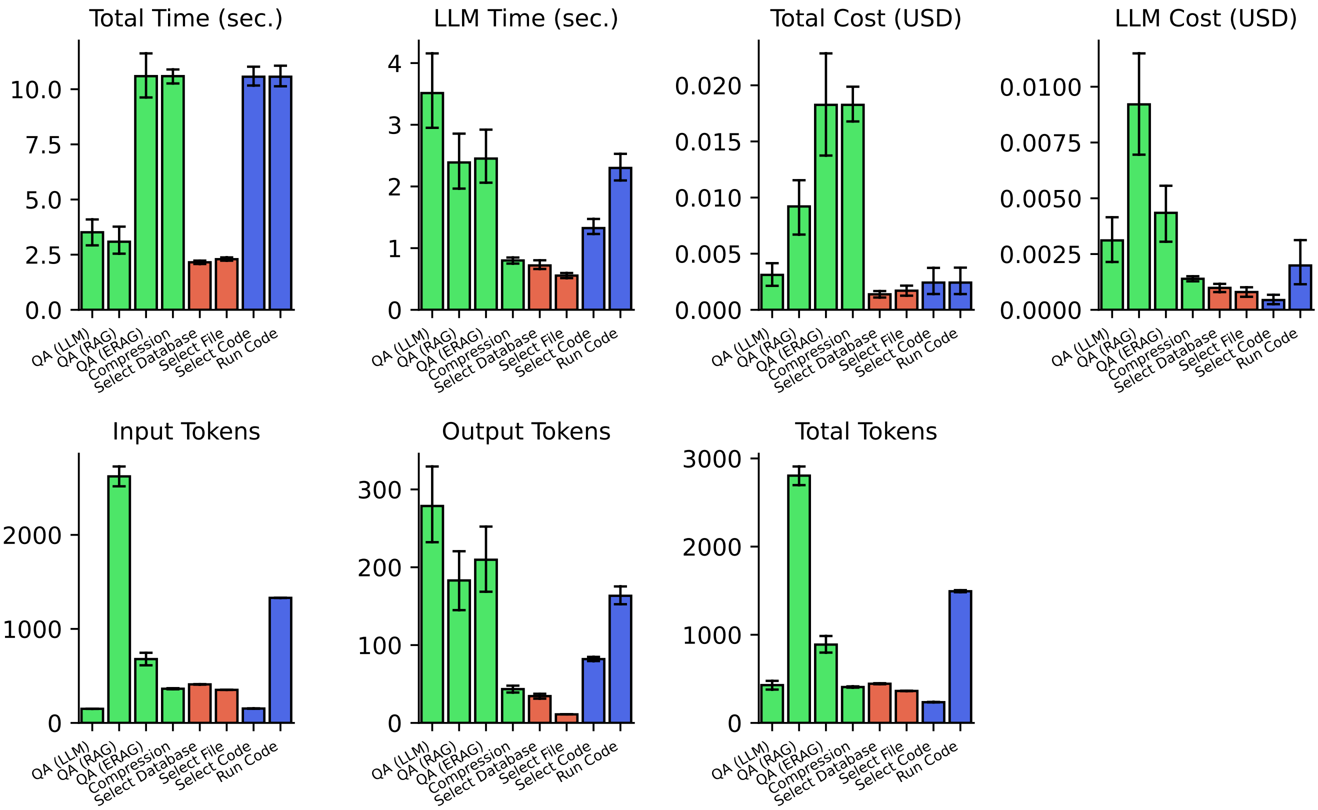


Fig. S1: Costs associated with the use of LLM in each module of BRAD are profiled. Each task is color-coded according to its respective tool module: Document Chat is represented in green, Software in red, and Database Search in blue.

**Table S2.** Summary of cost of different tasks performed by LLMs.

| Task | Model | Total Tokens | LLM Time (sec.) | Total Cost (USD, $\times 10^{-4}$) |
|---|---|---|---|---|
| QA (LLM) | `gpt-3.5-turbo-0125` | $303.14 \pm 57.22$ | $1.95 \pm 0.67$ | $0.0 \pm 0.0$ |
| | `gpt-4o` | $680.18 \pm 146.8$ | $6.65 \pm 1.71$ | $0.009 \pm 0.002$ |
| QA (RAG) | `gpt-3.5-turbo-0125` | $2746.5 \pm 340.04$ | $1.67 \pm 0.87$ | $0.001 \pm 0.0$ |
| | `gpt-4o` | $2863.77 \pm 367.62$ | $3.11 \pm 1.58$ | $0.017 \pm 0.003$ |
| QA (ERAG) | `gpt-3.5-turbo-0125` | $743.64 \pm 202.12$ | $1.5 \pm 0.57$ | $0.003 \pm 0.0$ |
| | `gpt-4o` | $1033.73 \pm 372.96$ | $3.41 \pm 1.35$ | $0.034 \pm 0.007$ |
| Compression | `gpt-3.5-turbo-0125` | $403.45 \pm 73.0$ | $0.73 \pm 0.49$ | $0.003 \pm 0.0$ |
| | `gpt-4o` | $410.78 \pm 77.89$ | $0.87 \pm 0.56$ | $0.034 \pm 0.007$ |
| Call code | `gpt-3.5-turbo-0125` | $1488.21 \pm 29.26$ | $2.17 \pm 0.52$ | $0.001 \pm 0.0$ |
| | `gpt-4o` | $1530.75 \pm 51.25$ | $3.22 \pm 0.74$ | $0.012 \pm 0.001$ |
| Select code | `gpt-3.5-turbo-0125` | $233.86 \pm 7.0$ | $1.25 \pm 0.16$ | $0.001 \pm 0.0$ |
| | `gpt-4o` | $243.5 \pm 14.15$ | $1.88 \pm 0.71$ | $0.012 \pm 0.001$ |
| Select database | `gpt-3.5-turbo-0125` | $448.38 \pm 29.29$ | $0.78 \pm 0.4$ | $0.0 \pm 0.0$ |
| | `gpt-4o` | $436.19 \pm 34.02$ | $0.59 \pm 0.23$ | $0.004 \pm 0.001$ |
| Select file | `gpt-3.5-turbo-0125` | $363.35 \pm 1.69$ | $0.47 \pm 0.13$ | $0.0 \pm 0.0$ |
| | `gpt-4o` | $361.25 \pm 1.65$ | $0.71 \pm 0.05$ | $0.004 \pm 0.0$ |
| **Average** | `gpt-3.5-turbo-0125` | 575.45 | 1.02 | 0.002 |
| | `gpt-4o` | 631.17 | 1.53 | 0.026 |

### 1.3. Document Chat Evaluation

We evaluated the performance of the RAG pipeline within the Document Chat module. Specifically, we measured Faithfulness and Answer Relevancy under two configuration settings of the tool module, comparing its performance to that of the LLM without the tool. We used metrics from the RAG Assessment (RAGAs) framework to benchmark the system [Es et al., 2023]:

**Faithfulness**. Faithfulness measures the factual consistency between the generated response and ground truth information taken from the literature database. Since this metric is entirely based on the literature, it is possible that the answer be factually incorrect while highly faithful to the literature database. It is measured by checking each of the generated claims is against the given context to determine if the generated response can be logically inferred from context. This is formalized as:

$$\text{Faithfulness} = \frac{\text{Number of Claims from Response inferred from Context}}{\text{Number of Claims from Response}},$$

where an LLM separate from the `Agent` determines the True/False positive/negative claims from each response relative to the retrieved quotations.

**Answer Relevance** Answer Relevance is a measure of how closely the generated response and given prompt are related. Answers that are incomplete or contain redundant information are typically assigned lower scores. We use the following formula to calculate answer relevance

$$\text{Answer Relevance} = \frac{1}{N}\left(\sum_{k=1}^{N} \frac{e_{g_k} \cdot e_o}{\|e_{g_k}\|\|e_o\|}\right),$$

where $N$ is the number of generated questions, $e_{g_k}$ is the vectorized embedding of the $k$th generated question from a generated response, and $e_o$ is the vectorized embedding of the original question.

**Experiment.** We obtained 500 documents totaling 18,000 pages, and curated over 100 research questions to evaluate our system. Each question was annotated as requiring knowledge from mathematics, computer science or biology, and the questions were further classified depending on the type of explanation required in the answer, i.e. simple, reasoning, or multicontext, according to the RAGAs framework.

We evaluated this dataset using three different `Agent`s. Each `Agent` can have its Document Chat module customized from the configuration file, in which the following fields can be set:

```
"num_articles_retrieved": <number of sources>,
"multiquery": <retrieval mechanism>,
"contextual_compression": <summary augmentation>,
"documentEnrichment": <expand with surrounding context augmentation>,
"rerank": <sorting mechanism during augmentation>,
"similarity": <retrieval mechanism>,
"mmr": <retrieval mechanism>,
"cut": <database control>
```

In this experiment, we evaluated two `Agents` that use the RAG pipeline with different configurations. Agent1 was configured to retrieve 10 documents based on their similarity to the question, and Agent2 was set to retrieve 10 documents based on a maximal marginal relevance (mmr) search and then apply contextual compression. Agent0 was set to have its Document Chat tool disabled and respond based only on the LLM. Each model used `gpt 4o-mini` from OpenAI as their LLM.

The performance of BRAD's RAG system is shown in fig. S2. This experiment demonstrates that the `Agent` can achieve improved performance by leveraging the Document Chat tool compared to using only the LLM (Agent0). Since the `Agent` is highly customizable and BRAD does not introduce a specific RAG methodology, we do not make quantitative claims about its performance relative to a standard LLM. Instead, the configuration of each `Agent`'s Document Chat tool—as well as other tools—should be tailored to the specific application for which it is deployed. Notably, the BRAD system is highly flexible, enabling deployment of complex RAG pipelines with minimal modifications to the configuration file.

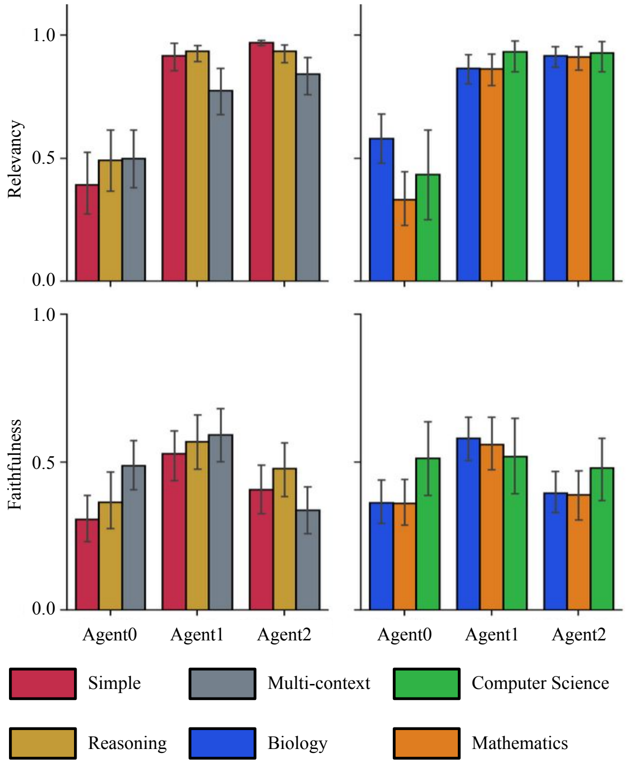


Fig. S2: **Document Chat Evaluation.** The Faithfulness and Relevancy of the responses from three `Agents` is shown for a dataset of computer science, mathematics, and biology questions. Agent1 and Agent2 both use the Document Chat tool with different configurations whereas Agent0 only uses the LLM.

### 1.4. Comparison with Mainstream Tools

Table table S3 compares the BRAD system with other mainstream agentic systems. The systems are evaluated across three key categories:

(1) **Deployment and Access**: This category assesses how the system is used, including its intended use case, programming requirements, whether it can be run locally, and whether it is open-source.
(2) **Tools**: This category focuses on the system's ability to interact with external data sources, including internet search capabilities, user data retrieval, search control, and integration with custom software tools.
(3) **Scientific Rigor**: This category examines the system's suitability for research applications and workflows as a research tool.

While category (1) influences how easily a system can be adopted, categories (2) and (3) are essential for integrating an agentic system into a research environment without requiring complete human oversight. Many commercial systems fall short in these areas, offering minimal control over how they interact with research tools and resources, as well as limited transparency for validating their outputs.

In contrast, most academic systems excel in terms of scientific rigor, particularly regarding reproducibility, and provide a high degree of control over how the agentic systems interact with specific research tools. However, because many academic agentic systems are designed with a narrow scope—typically to solve a single task such as writing a report—they lack the flexibility to adapt their advantages to broader or different problems. While many of these systems require programming knowledge, these systems are mostly straightforward to deploy.

Frameworks for building agentic systems provide both a high degree of flexibility and seamless integration with research tools. They also offer the potential for users to implement rigorous reproducibility within the agentic systems. Unlike the more specialized academic and commercial systems listed here, which are designed for discrete use cases, these frameworks serve a broader purpose as they form the foundation for a wide range of agentic systems. However, leveraging these frameworks effectively requires strong programming knowledge, making them less accessible to non-technical users, which need to implement their own reproducibility or record keeping systems.

In contrast, the BRAD system combines the best qualities of these categories, being accessible to non-technical users while offering the flexibility to integrate various tools and features. At the same time, it maintains a high level of scientific rigor, ensuring it meets the standards required for research applications.

<table>
<tr><th colspan="3">System Type</th><th colspan="4">Deployment & Access</th><th colspan="4">Tools</th><th colspan="3">Scientific Rigor</th></tr>
<tr><th></th><th colspan="2"></th><th>Use Case</th><th>Programming requirements</th><th>Ability to Run Locally</th><th>Open Source</th><th>Search Internet</th><th>Search User Data</th><th>Control over Search</th><th>Custom Software Tools</th><th>Reproducible</th><th>Traceable</th><th>Data privacy</th></tr>
<tr><td rowspan="7">Commercial</td><td>ChatGPT</td><td>[OpenAI, 2024a]</td><td rowspan="5">General Purpose</td><td rowspan="7">None</td><td rowspan="7">No</td><td rowspan="7">No</td><td rowspan="6">Yes</td><td rowspan="7">Yes</td><td rowspan="7">No</td><td rowspan="7">No</td><td rowspan="7">Low</td><td rowspan="7">Low</td><td rowspan="7">Low</td></tr>
<tr><td>PerplexityAI</td><td>[AI, 2024]</td></tr>
<tr><td>Claude</td><td>[Anthropic, 2024]</td></tr>
<tr><td>Gemini</td><td>[DeepMind, 2024a]</td></tr>
<tr><td>DeepSeek</td><td>[DeepSeek, 2024]</td></tr>
<tr><td>Deep Research</td><td>[OpenAI, 2025]</td><td rowspan="2">Research</td></tr>
<tr><td>NotebookLM</td><td>[DeepMind, 2024b]</td><td>No</td></tr>
<tr><td rowspan="7">Academic</td><td>Kragen</td><td>[Matsumoto et al., 2024]</td><td rowspan="7">Individual Research Tasks</td><td rowspan="7">Low</td><td rowspan="6">Yes</td><td rowspan="6">Yes</td><td>No</td><td>Yes</td><td>No</td><td>No</td><td rowspan="6">High</td><td rowspan="7">High</td><td rowspan="7">High</td></tr>
<tr><td>LmRAC</td><td>[Craig and Drăghici, 2024]</td><td>Yes</td><td>Yes</td><td>No</td><td>Yes</td></tr>
<tr><td>ESCARGOT</td><td>[Matsumoto et al., 2025]</td><td>No</td><td>Yes</td><td>Yes</td><td>Yes</td></tr>
<tr><td>Virtual Lab</td><td>[Swanson et al., 2024]</td><td>No</td><td>No</td><td>Yes</td><td>Yes</td></tr>
<tr><td>Coscientist</td><td>[Boiko et al., 2023]</td><td>Yes</td><td>Yes</td><td>Yes</td><td>Yes</td></tr>
<tr><td>BioDiscovery</td><td>[Roohani et al., 2024]</td><td>Yes</td><td>Yes</td><td>Yes</td><td>No</td></tr>
<tr><td>Storm</td><td>[Shao et al., 2024]</td><td>No</td><td>No</td><td>Yes</td><td>No</td><td>No</td><td>No</td><td>Low</td></tr>
<tr><td rowspan="5">Frameworks</td><td>LangChain</td><td>[Affarth, 2023]</td><td rowspan="5">Building Agentic Systems</td><td rowspan="5">High</td><td rowspan="5">Yes</td><td rowspan="5">Yes</td><td rowspan="5" colspan="4">Possible</td><td rowspan="5" colspan="3">Possible</td></tr>
<tr><td>CrewAI</td><td>[CrewAI, 2024]</td></tr>
<tr><td>AutoGen</td><td>[Wu et al., 2023]</td></tr>
<tr><td>LlamaIndex</td><td>[Liu, 2022]</td></tr>
<tr><td>Swarm</td><td>[OpenAI, 2024b]</td></tr>
<tr><td></td><td colspan="2">BRAD</td><td>General Research</td><td>Low</td><td>Yes</td><td>Yes</td><td>Yes</td><td>Yes</td><td>Yes</td><td>Yes</td><td>High</td><td>High</td><td>High</td></tr>
</table>

**Table S3. Comparison of LLM Applications and Frameworks.** This table presents a comparative analysis of various LLM-based applications based on key features relevant to scientific workflows. The features include: *Use Case* – Whether the tool addresses a specific research need or supports a broader range of applications. *Programming Requirements* – The level of technical expertise needed to use the tool. *Ability to Run Locally* – Whether the tool can be executed on a user's machine. *Open Source* – The transparency of the tool's source code. *Internet Search* – The capability to search online sources and databases. *User Data Search* – The ability to access and query local user data and databases. *Control Over Search* – The extent to which users can configure and direct searches. *Custom Tools* – Support for integration with user-defined tools. *Reproducibility* – Whether the tool produces consistent and repeatable outputs. *Traceability* – The ability to track reasoning and data usage. *Data Privacy* – Whether the system requires users to share their data.

## References


B. Affarth. *Generative AI with LangChain: Build Large Language Model (LLM) Apps with Python, ChatGPT, and Other LLMs*. Packt Publishing Ltd., Birmingham, UK, 2023. ISBN 978-1-83508-346-8.

P. AI. Perplexity ai: Ai-powered search and chat. `https://www.perplexity.ai`, 2024. Accessed: 2025-02-14.

Anthropic. Claude: Constitutional ai by anthropic. `https://www.anthropic.com`, 2024. Accessed: 2025-02-14.

M. Ashburner, C. A. Ball, J. A. Blake, D. Botstein, H. Butler, J. M. Cherry, A. P. Davis, K. Dolinski, S. S. Dwight, J. T. Eppig, et al. Gene ontology: tool for the unification of biology. *Nature genetics*, 25(1):25–29, 2000.

D. A. Boiko, R. MacKnight, B. Kline, and G. Gomes. Autonomous chemical research with large language models. *Nature*, 624(7992): 570–578, 2023.

H. Chen, J. Chen, L. A. Muir, S. Ronquist, W. Meixner, M. Ljungman, T. Ried, S. Smale, and I. Rajapakse. Functional organization of the human 4d nucleome. *Proceedings of the National Academy of Sciences*, 112(26):8002–8007, 2015.

T. T. S. Consortium*, R. C. Jones, J. Karkanias, M. A. Krasnow, A. O. Pisco, S. R. Quake, J. Salzman, N. Yosef, B. Bulthaup, P. Brown, et al. The tabula sapiens: A multiple-organ, single-cell transcriptomic atlas of humans. *Science*, 376(6594):eabl4896, 2022.

D. B. Craig and S. Drăghici. Lmrac: a functionally extensible tool for llm interrogation of user experimental results. *Bioinformatics*, page btae679, 2024.

CrewAI. Crewai: Ai agent collaboration framework. `https://www.crewai.com/`, 2024. Accessed: 2025-02-14.

G. DeepMind. Gemini: Multimodal ai by google deepmind. `https://deepmind.google/technologies/gemini`, 2024a. Accessed: 2025-02-14.

G. DeepMind. Notebooklm: Ai-powered research assistant. `https://notebooklm.google`, 2024b. Accessed: 2025-02-14.

DeepSeek. Deepseek: Ai-powered search and language model. `https://www.deepseek.com`, 2024. Accessed: 2025-02-14.

S. Es, J. James, L. Espinosa-Anke, and S. Schockaert. Ragas: Automated evaluation of retrieval augmented generation. *arXiv preprint arXiv:2309.15217*, 2023.

Y. Gao, Y. Xiong, X. Gao, K. Jia, J. Pan, Y. Bi, Y. Dai, J. Sun, and H. Wang. Retrieval-augmented generation for large language models: A survey. *arXiv preprint arXiv:2312.10997*, 2023.

M. Kanehisa and S. Goto. Kegg: kyoto encyclopedia of genes and genomes. *Nucleic acids research*, 28(1):27–30, 2000.

M. V. Kuleshov, M. R. Jones, A. D. Rouillard, N. F. Fernandez, Q. Duan, Z. Wang, S. Koplev, S. L. Jenkins, K. M. Jagodnik, A. Lachmann, et al. Enrichr: a comprehensive gene set enrichment analysis web server 2016 update. *Nucleic acids research*, 44(W1): W90–W97, 2016.

V. Levenshtein. Binary codes capable of correcting deletions, insertions, and reversals. *Proceedings of the Soviet physics doklady*, 1966.

P. Lewis, E. Perez, A. Piktus, F. Petroni, V. Karpukhin, N. Goyal, H. Küttler, M. Lewis, W.-t. Yih, T. Rocktäschel, et al. Retrieval-augmented generation for knowledge-intensive nlp tasks. *Advances in Neural Information Processing Systems*, 33:9459–9474, 2020.

J. Liu. Llamaindex, November 2022. URL `https://github.com/jerryjliu/llama_index`. If you use this software, please cite it as below.

L. Luebbert and L. Pachter. Efficient querying of genomic reference databases with gget. *Bioinformatics*, 39(1):btac836, 2023.

N. Matsumoto, J. Moran, H. Choi, M. E. Hernandez, M. Venkatesan, P. Wang, and J. H. Moore. Kragen: a knowledge graph-enhanced rag framework for biomedical problem solving using large language models. *Bioinformatics*, 40(6), 2024.

N. Matsumoto, H. Choi, J. Moran, M. E. Hernandez, M. Venkatesan, X. Li, J.-H. Chang, P. Wang, and J. H. Moore. Escargot: an ai agent leveraging large language models, dynamic graph of thoughts, and biomedical knowledge graphs for enhanced reasoning. *Bioinformatics*, 41(2):btaf031, 2025.

OpenAI. Chatgpt: Large language model by openai. `https://openai.com/chatgpt`, 2024a. Accessed: 2025-02-14.

OpenAI. Swarm: Multi-agent coordination by openai. `https://github.com/openai/swarm`, 2024b. Accessed: 2025-02-14.

OpenAI. Introducing deep research, February 2025. URL `https://openai.com/index/introducing-deep-research/`. Accessed: 2025-02-18.

J. Pickard, C. Stansbury, A. Surana, L. Muir, A. Bloch, and I. Rajapakse. Dynamic sensor selection for biomarker discovery. *arXiv preprint arXiv:2405.09809*, 2024.

Y. Roohani, A. Lee, Q. Huang, J. Vora, Z. Steinhart, K. Huang, A. Marson, P. Liang, and J. Leskovec. Biodiscoveryagent: An ai agent for designing genetic perturbation experiments. *arXiv preprint arXiv:2405.17631*, 2024.

P. Sahoo, A. K. Singh, S. Saha, V. Jain, S. Mondal, and A. Chadha. A systematic survey of prompt engineering in large language models: Techniques and applications. *arXiv preprint arXiv:2402.07927*, 2024.

M. Schaefer, P. Peneder, D. Malzl, M. Peycheva, J. Burton, A. Hakobyan, V. Sharma, T. Krausgruber, J. Menche, E. M. Tomazou, et al. Multimodal learning of transcriptomes and text enables interactive single-cell rna-seq data exploration with natural-language chats. *bioRxiv*, pages 2024–10, 2024.

Y. Shao, Y. Jiang, T. A. Kanell, P. Xu, O. Khattab, and M. S. Lam. Assisting in writing wikipedia-like articles from scratch with large language models. *arXiv preprint arXiv:2402.14207*, 2024.

Y. Shavit, S. Agarwal, M. Brundage, S. Adler, C. O'Keefe, R. Campbell, T. Lee, P. Mishkin, T. Eloundou, A. Hickey, et al. Practices for governing agentic ai systems. *Research Paper, OpenAI, December*, 2023.

K. Swanson, W. Wu, N. L. Bulaong, J. E. Pak, and J. Zou. The virtual lab: Ai agents design new sars-cov-2 nanobodies with experimental validation. *bioRxiv*, pages 2024–11, 2024.

J. Wei, X. Wang, D. Schuurmans, M. Bosma, F. Xia, E. Chi, Q. V. Le, D. Zhou, et al. Chain-of-thought prompting elicits reasoning in large language models. *Advances in neural information processing systems*, 35:24824–24837, 2022.

H. Weintraub. Summary: genetic tinkering—local problems, local solutions. In *Cold Spring Harbor Symposia on Quantitative Biology*, volume 58, pages 819–836. Cold Spring Harbor Laboratory Press, 1993.

Q. Wu, G. Bansal, J. Zhang, Y. Wu, S. Zhang, E. Zhu, B. Li, L. Jiang, X. Zhang, and C. Wang. Autogen: Enabling next-gen llm applications via multi-agent conversation framework. *arXiv preprint arXiv:2308.08155*, 2023.
